# Depression in nursing students during the COVID-19 pandemic: Systematic review and meta-analysis

**DOI:** 10.1371/journal.pone.0304900

**Published:** 2024-07-24

**Authors:** Carmen Quesada-Puga, Gustavo R. Cañadas, José Luis Gómez-Urquiza, Raimundo Aguayo-Estremera, Elena Ortega-Campos, José Luis Romero-Béjar, Guillermo A. Cañadas-De la Fuente

**Affiliations:** 1 University Hospital Torrecardenas, Andalusian Health Service, Almería, Spain; 2 Department of Didactic of Mathematics, Faculty of Education Science, University of Granada, Granada, Spain; 3 Faculty of Health Sciences, University of Granada, Ceuta, Spain; 4 Department of Psychobiology and Methodology in Behavioral Sciences, Complutense University of Madrid, Pozuelo de Alarcón, Spain; 5 Department of Psychology, University of Almería, Almería, Spain; 6 Department of Statistics and Operations Research, University of Granada, Granada, Spain; 7 Instituto de Investigación Biosanitaria (ibs.GRANADA), Granada, Spain; 8 Institute of Mathematics of the University of Granada (IMAG), Granada, Spain; 9 Faculty of Health Sciences, University of Granada, Granada, Spain; 10 Brain, Mind and Behaviour Research Center (CIMCYC), University of Granada, Granada, Spain; University of Huelva: Universidad de Huelva, SPAIN

## Abstract

The pandemic meant a change in academic approach. This had an impact on the mental health of students, leading to, among other problems, depressive disorders. The aim of this study was to find out the prevalence and factors that favoured the development of depression in nursing students during the COVID-19 pandemic. A systematic review with meta-analysis of prevalence was conducted in October 2023, using Pubmed, CINAHL and Scopus as the data sources used for the search. This review followed the guidelines outlined in the Preferred Reporting Items for Systematic Reviews and Meta-Analyses (PRISMA). Search equation was: “(undergraduate nurses OR nursing students) AND depression AND (COVID-19 OR Sars-CoV-2)”. The final set of articles was N = 12. Quantitative primary studies using anonymous scales and surveys to assess the prevalence of depression in nursing students in the last 3 years were included. Studies show a high prevalence of depression among young university students with figures above 50%. The total sample of students in the meta-analysis was n = 4,479 with a prevalence value of 32% (CI95% 22%-42%). Affected students are characterised by young, female students. Concerns included generalised academic uncertainty, social isolation, work overload, fear of contagion and concern about teaching delivery. Coping mechanisms were generally resilience, spiritual support, laughter therapy, seeking information about COVID-19 and eating food. In conclusion, students, especially female students, are at high risk of depression due to social isolation. In addition, coping techniques were inadequate and future strategies to prevent this situation should be considered.

## 1. Introduction

According to the World Health Organisation (WHO), depression is a mental disorder characterised by low mood or a loss of interest in or pleasure from activities over a prolonged period of time. It can affect anyone and is the consequence of a complex interaction of social, psychological and biological factors [1]. It has an impact on the general level of activities of the affected person, is quite common, is emotionally costly and disabling, and is therefore a serious public health problem worldwide [2]. This disorder affects 4.4% of the entire population, i.e. some 300 million people worldwide. It can also cause great suffering for those affected, even leading to suicide. In fact, suicide is the fourth leading cause of death in the 15–29 age group [3, 4]. Treatment of this disorder is currently a major challenge. In non-developed countries, more than 75% of those affected do not receive treatment [5] and it is currently one of the leading causes of disability in developing countries [6]. In fact, in the USA alone, the cost to society of this disorder is $210 billion [7]. Signs and symptoms that stand out in this syndrome are often altered state of mind, psychomotor changes, loss of energy, appetite disorders, sleep disturbances, concentration problems and even suicidal ideation, among others [8, 9]. Depression manifests in both sexes, regardless of age or social class, although the literature reports that women are more prone to depression, mainly due to hormonal problems [10]. However, it is more common for the first episode to occur in adolescence during secondary school. This is usually due to academic pressure and family-related factors. All this leads to poor academic achievement and interpersonal problems [11] which, if left unresolved, tend to worsen with age, especially in women [12].

University students are no exception. The transition to secondary school is a major change and a stressful event in itself. After overcoming the problems of high school, they face a major change and therefore an even more stressful situation at university [13]. This stress significantly affects students, leading to anxiety problems, poor sleep quality and insomnia [14, 15], and consequently to depression [16]. Moreover, these physical and psychological problems become chronic, diminishing the student’s quality of life [17] and if they do not have a good support network, hopelessness and finally suicidal ideas appear [18]. In addition, there are other risk factors for depression such as relationship with parents, history of physical/sexual abuse, living outside the family home, toxic habits or sleeping problems, among others [19]. Health professionals have a stressful job. Working conditions are not always good, as they are subjected to a lot of pressure and sometimes even aggression [20]. This, together with other problems such as lack of sleep and work overload, causes professionals, and more specifically nurses, to suffer from depression [21] and Burnout syndrome [22–24].

Due to the global COVID-19 pandemic, most academic institutions were forced to adopt the use of virtual teaching and learning methods to try to save the academic year. This sudden change in teaching method left students dissatisfied with their learning experience and resulted in stressful work overloads that began to generate more symptoms of anxiety and depression [25]. In fact, recent meta-analytical studies claim that depression in this group was as high as 52% [26]. Given that university students in the health professions are in training, the problem is further exacerbated. In addition to the academic pressure, there are the clinical internships, where students start to come into contact with patients. Inexperience and dealing with sick people becomes a very stressful situation over time, leading to anxiety, loss of confidence and decreased academic performance [27]. Therefore, such academic stress is associated with a deterioration of psychosocial well-being and eventually leads to depression [28, 29]. Finally, nursing students who faced the COVID-19 pandemic suffered particularly badly. Confinement was a very hard situation for the university population [30] but the future nurses also went through changes in the way they learned, the exposure during their clinical practice and their individual coping [31]. This situation was further aggravated if they had infected family members and/or were afraid of the pandemic [32].

The need for this study is justified in case a similar situation arises in the future. The knowledge gained would help to foresee this disorder in university nursing students. Therefore, the aim of this study was to analyse the factors that favoured the development of depression in university nursing students during the COVID-19 pandemic, as well as to estimate the prevalence of this disorder in this university group.

## 2. Methodology

A systematic review of the scientific literature and a meta-analysis of prevalence was carried out for this article.

### 2.1 Search strategy

The electronic databases used for the bibliographic search were: Pubmed, CINAHL and Scopus in accordance with the recommendations made in the declaration Preferred Reporting Items for Systematic Reviews and Meta-Analyses (PRISMA) [33]. The following search equation was used: “(undergraduate nurses OR nursing students) AND depression AND (COVID-19 OR Sars-CoV-2)”. The descriptors of the search equation were extracted through the thesaurus Medical Subject Heading (MeSH) and its equivalent in Spanish. The search was conducted in October 2023.

### 2.2 Study selection

The following inclusion and exclusion criteria were used to select the articles:

Inclusion criteria: primary quantitative studies; the articles that were included were in English and Spanish; the design had to be quantitative; without geographical restriction by country; the study population of the articles was focused on nursing students suffering from depression at the time of COVID-19; and finally, they had to have been published in the last 3 years, i.e. between 2020 and 2023 which is when the COVID-19 pandemic took place.

Exclusion criteria: those where the COVID-19 pandemic was not present, as well as doctoral theses and articles with unreliable information, or without access to the full text.

Once the aforementioned search criteria had been applied, the bibliography was selected in the following steps: reading the title, reading the abstract, reading the full text and finally, reviewing the references included in the selected articles.

### 2.3 Variables and data collection

A data collection notebook was created including: authorship, year of publication, methodological design, prevalence, main results and classification according to the level of scientific evidence.

The review and selection of articles was carried out by two reviewers. In case of disagreement, a third reviewer would act.

### 2.4 Critical reading (risk of bias) and scientific evidence

Studies critical reading was performed following the Mixed Methods Appraisal Tool (MMAT) guidelines [34], so that any potential biases and limitations could be discussed. The levels of evidence and grades of recommendation were awarded according to those established by the Oxford Center for Evidence-Based Medicine (OCEBM) [35, 36].

### 2.5 Data analysis

A random-effects meta-analysis was performed using the meta-analysis package of Statsdirect software to estimate the prevalence of depression in nursing students. To estimate the prevalence, the total sample size and the sample with depression of each study were used. Heterogeneity was calculated using the I2 index and publication bias using Egger’s test.

## 3. Results

### 3.1 Characteristics of the studies

**Fig 1:**
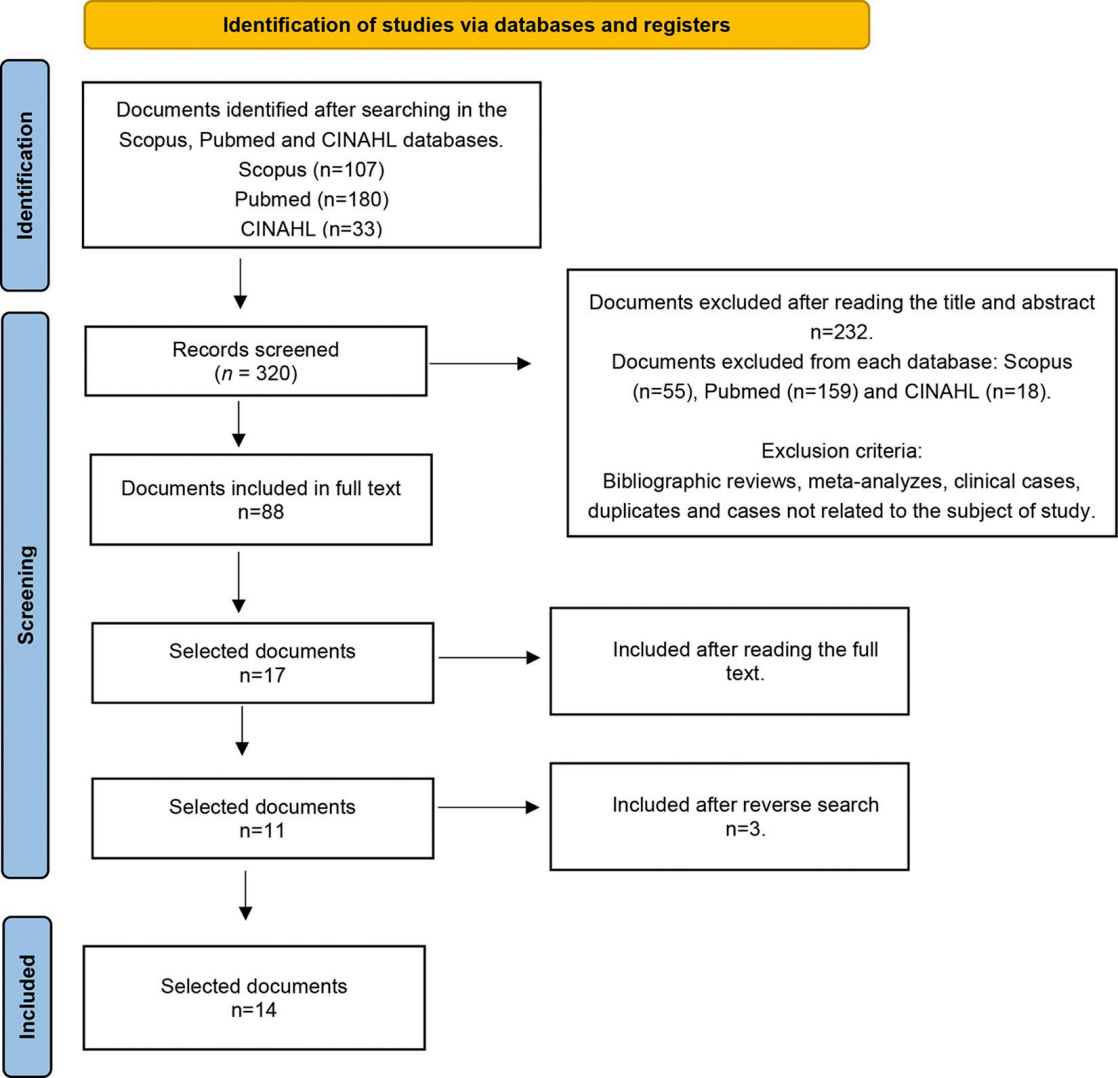
Flow diagram of the search process.

In the initial search of the databases described above, 320 articles were selected. After applying the inclusion and exclusion criteria, a screening was performed and 88 published papers were selected. No duplicate articles were found. After reading the title and abstract, 37 articles were selected and after a full reading, 11 were left. Finally, by performing a reverse search analysing the bibliography of these 11 articles, 3 more were retrieved, leaving a final sample of n = 14 articles. The selection diagram repre-senting the search and selection process is shown in Fig 1.

All the studies selected were cross-sectional studies, with the exception of one of them which was a case-control study. The sample was selected from nursing students, some of them during their internships. The countries where the studies were carried out were the USA, Albania, Greece, Jordan, Korea, Spain, Turkey, Italy, New South Wales, Vietnam and Saudi Arabia.

Studies used health and life satisfaction scales, depression scales, anxiety scales and other questionnaires to measure the level of depression. Examples of scales used were the Hamilton Rating Scale for Depression (HRSD), the Montgomery Asberg Depression Rating Scale (MADRS) or the Patient Health Questionnaire (PHQ-9).

Table 1 shows the most relevant data related to the different studies and the characteristics of each one [37–50] and S1 Table shows the results of MMAT checklist critical reading [34].

**Table 1:** Information on the selected studies, basic characteristics and results.

AUTHOR AND YEAR (COUNTRY)	DESIGN	SAMPLE	INSTRUMENTS	PREVALENCE	RESULTS	LE/ GR	QUALITY
Alomari et al. [46], 2021 (New South Wales).	Cross-sectional	178 nursing students.	Interview with open and closed questions, and Beck Depression Inventory	-	Need for academic support, preparation for online classes, clinical practice and clinical placement. In addition, they presented financial needs, social isolation, lack of communication and preparation for e-learning.	2c / B	Medium
Alsolais et al. [45], 2021 (Saudi Arabia).	Cross-sectional	1057 nursing students.	DASS-21 COPE	9% had some degree of depression, anxiety and stress.	The main causes were perceived severity by COVID-19, risk of infection, knowing someone infected and fear. For depression, 56.7% of respondents were categorized as in the normal range, 8.3% were severe, and 11.0% were very severe. The models for depression (F[30, 461] = 4.92, p < 0. 001), anxiety (F[30, 461] = 5.84, p < 0.001), and stress (F[30, 461] = 4.86, p < 0.001) were statistically significant, explaining 19.3% (R2 = 0.243, adjusted R2 = 0.193), 22.8% (R2 = 0.275, adjusted R2 = 0.228) and 19.1% (R2 = 0.240, adjusted R2 = 0.191) of the variance in depression, anxiety, and stress, respectively.	2c / B	Medium
Kells and Jennings [44], 2023 (United States of American).	Cross-sectional	161 nursing students.	PHQ-9	19.8% reported depression.	The scores were: 56.6% stress, 68.2% felt overwhelmed, 18.7% anxiety and 55.6% worried about the future. 54.4% reported that the pandemic influenced their interest in nursing. PHQ-9 scores indicated that 77% of the students (n = 124) did not suffer from depression.	2c / B	Medium
Kim et al. [38], 2021 (United States of American).	Cross-sectional	173 nursing students.	PHQ-9	More than 50% reported symptoms of moderate or severe depression.	High family functioning decreased the risk of depression (OR = 0.41). Spiritual support decreased the risk of depression (OR = 0.48). Compared with the pre-lockdown period, students reported moderate-to-severe depression (median [IQR]; 3 [1, 6] vs. 10 [4, 15]; p < 0.001) during the lockdown. Similarly, more students experienced moderate-to-severe depression (12.9% vs. 51.5%) during the lockdown. However, spiritual support was associated with a reduction of depression by half.	2c / B	High
Kwon [42], 2023 (Korea)	Cross-sectional	301 nursing students.	PHQ-9	33.2% of students reported depression and 29.2% anxiety.	Depression was positively correlated with anxiety and negatively correlated with pro-health behaviors.	2c / B	Medium
Losa-Iglesias et al. [47], 2023 (Spain)	Cases-controls	140 nursing students.	Beck Depression Inventory	62.8% reported depression.	Virtual teaching was associated with higher levels of depression.		
Mendez-Pinto et al. [49], 2023 (Spain)	Cross-sectional	304 nursing students.	DASS-42	15.5% presented depression, 26.7% post-traumatic stress and 39.8% anxiety.	Among the risk factors were being a woman and cohabitation of the students.	2c / B	Medium
Mosteiro-Diaz et al. [48], 2023 (Spain)	Cross-sectional	1319 nursing students.	HADS	38.2% showed symptoms of depression.	Depressive symptoms were increased by toxic habits, feelings of fear, having a close person infected, and stress.	2c / B	High
Nguyen et al. [43], 2023 (Vietnam)	Cross-sectional	191 nursing students.	GAD-7	21.5% presented depressive symptoms.	Almost half of the students (49.2%) who presented depression did not have strategies to cope with mental problems.	2c / B	Medium
Nihal Bostanci et al. [37], 2021 (Turkey).	Cross-sectional	787 nursing students.	DASS-21 and GAD-7	50% reported depression and anxiety.	Need for psychological support programmes for nursing students during COVID-19.	2c / B	Medium
Ozturk and Tekkas- Kerman [50], 2022 (Turkey).	Randomised controlled trial	61 nursing students.	DASS-42 DJGLS	-	The laughter therapy group scored significantly lower on depression than the control group. There was no statistically significant difference between the groups in terms of depression (t = 0.854, p = 0.960), anxiety (t = 1.563, p = 0.123) and stress (t = 1.128, p = 0.264) before laughter therapy. However, mean depression subscale scores in the intervention group after online laughter therapy were lower than before the intervention. Also the results of the independent t-test showed a significant difference between the intervention and control groups (t = -2.997, p = 0.003).	1c / A	Medium
Patelarou et al. [40], 2021 (Albania, Greece and Spain)	Cross-sectional	787 nursing students from Greece (348), Spain (242) and Albania (197).	PHQ-9	33% were depressed. This was highest in Spaniards with 59.1%, followed by Albania with 34.5% and Greeks with 21.8%.	Lower age was associated with increased depression due to the impact of confinement and quarantine. The levels of depression were higher for Spanish students than for Greek and Albanian students (p < 0.001). The prevalence of mild depression was 31%, of severe depression was 6%, of moderate-severe depression was 13.3% and of moderate depression was 17.2%. The sample of students who experienced minimal or no depression was 32.5%.	2c / B	High
Rayan [41], 2023 (Jordan)	Cross-sectional	224 nursing students.	DASS-21	30.1% reflected symptoms of depression.	Depression was associated with online teaching, academic load, and the possibility of technological failures such as internet connection during an exam.	2c / B	Medium
Urban et al. [39], 2022 (United States of American).	Cross-sectional	192 nurses in internships.	PHQ-9 and GAD-7	31.2%.	31.2% reported depression, 76% reported stress and 27.6% anxiety. However, 79% referred to themselves as resilient.	2c / B	High

COPE: Coping Orientation of Problem Experienced; DASS-21: Depression, Anxiety and Stress Scale short-form version (21 items); DASS-42: Depression, Anxiety and Stress Scale (42 items); DJGLS: De Jong Gierveld Loneliness Scale; GAD-7: The General Anxiety Disorder-7; Hospital Anxiety and Depression Scale: HADS; LE: Level of Evidence; GR: Level of Recommendation; PHQ-9: Patient Health Questionnaire (9 items).

### 3.2 Prevalence of depression in nursing students and meta-analytical estimation

The shared aim of the studies was to find out how the COVID-19 pandemic, the quarantine and the change in the way of teaching had affected the mental health of nursing students. Mainly depression and strategies to prevent it were assessed, but data were also collected on anxiety, isolation and feelings of loneliness perceived by the students. For this purpose, data were taken from individual surveys completed anonymously by the students [38, 46, 47, 49].

The prevalence found was, in general, quite high, exceeding 50% [37, 38], more than 30% [39–42], around 20% [43, 44] and 9% [45]. In Spain, the levels of depression reached by students were 59.1% [46], 62.8% [47], 38.2% [48] and 15.5% [49] a figure that was surprisingly high compared to students from other countries such as Albania or Greece [40].

Comparing previous levels of depression in nursing students, some of them had moderate levels of depression before the pandemic, which evolved into severe depression during confinement [37].

Prior to the pandemic, anxiety and stress levels were moderate and became severe with confinement (p < 0.001). Also, high resilience was negatively associated with moderate-to-severe depression (OR = 0.50; 95%CI = 0.26–0.95; p = 0.036). Similarly, high family functioning was negatively associated with moderate-to-severe depression (OR = 0.41; 95%CI = 0.20–0.81; p = 0.011) [38].

**Fig 2:**
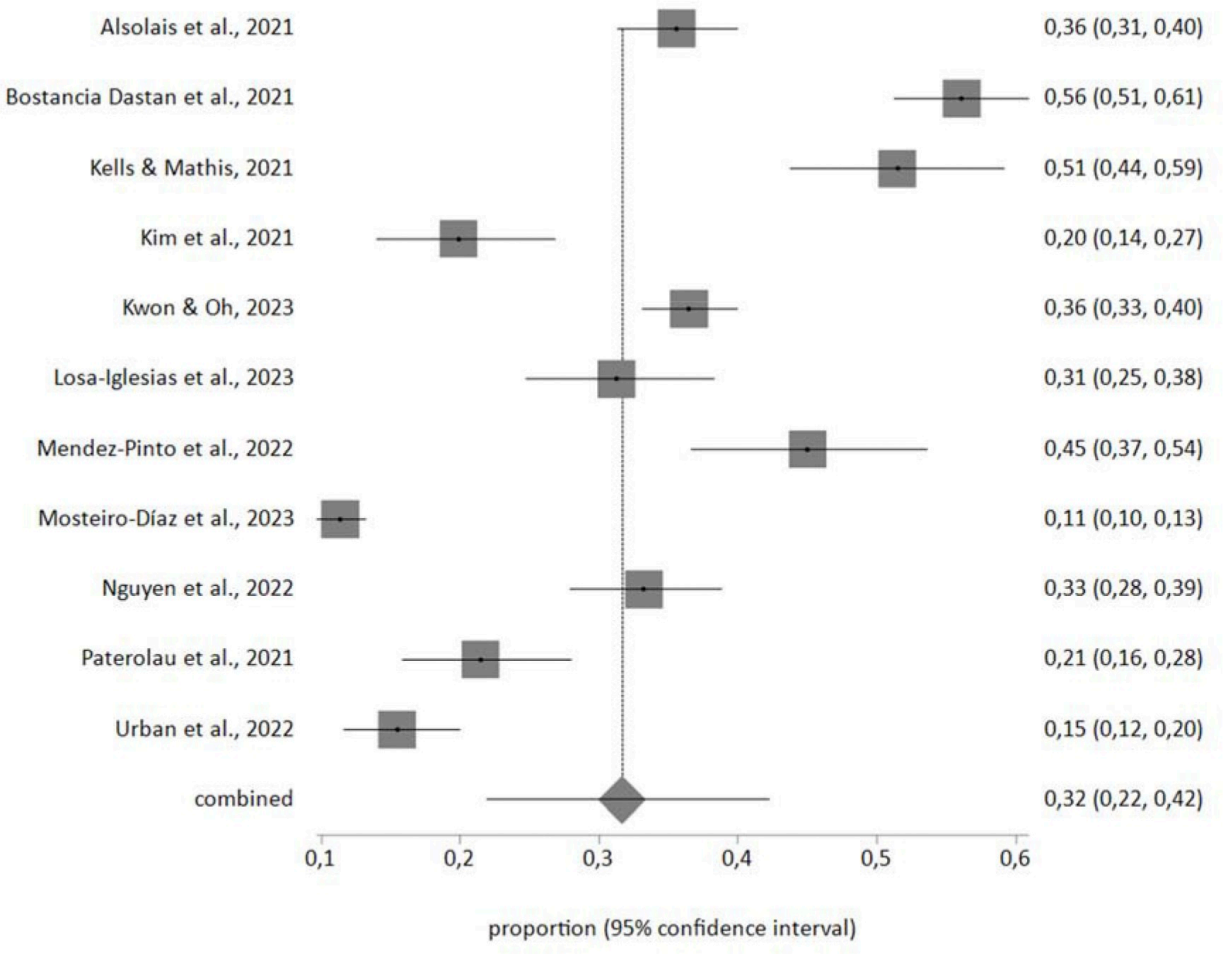
Foresplot of the prevalence of depression in nursing students during COVID.

A meta-analysis of the prevalence of depression in nursing students during the pandemic was conducted with the studies that included the necessary information (n = 11). The samples affected by depression in each study, independently of the scale, were included in the meta-analysis. The total sample of students in the meta-analysis was n = 4,479 with a prevalence value of 32% (CI95% 22%-42%). The forestplot is shown in Fig 2. The I2 value was 98% and Egger’s test value showed no publication bias.

### 3.3 Characteristics of university nursing students

Common respondent characteristics were observed in the study by Kim et al. [38], where the sample was 173 undergraduate nursing students. 93.1% were female and over 57% were white persons. Fourteen percent had worked with COVID-19 patients and 75% had experienced quarantine or isolation of some kind.

These characteristics are very similar in all the studies, which seems to indicate that the nursing profession is largely practised by white women [37, 38, 40, 44, 45, 50]. For example, in the study by Kells et al. [44] 94.4% of the participants were white heterosexual women aged 18–21 years.

In the study by Mendez-Pinto et al. [49], a sample of 304 nursing students was taken, of which 266 were women and 38 men. The prevalence of depression was 15.5%, 26.7% reported post-traumatic stress and 39.8% reported anxiety. Depression mainly affected women, representing 16.5%, while that of men was 1.1%. Therefore, being a woman was considered a risk factor for suffering from depressive symptoms.

It is remarkable and noteworthy that decreasing age was associated with an increase in depression, i.e. younger students are more at risk of depression [40].

### 3.4 Concerns of nursing students

Several authors state that the main concern of students was the disruption of their classroom-based studies. The move to a telematic form of education and the difficulty in participating in class makes it very difficult to manage academic matters. Depression was associated with distance teaching, academic load, delay in completing work, the possibility of Internet connection failure while taking an exam or interruption during learning. Examples of this would be being able to meet deadlines for assignments, as well as coming to internship with little experience in dealing with patients. If these patients had COVID-19 the situation was more stressful, as the continuous exposure was a risk of contagion for all of them. The students’ perception of contracting COVID-19 was high and this caused fear. Therefore, providing students with clear guidelines for infection prevention helped them feel more confident in the classroom and in clinical practice. It was also key to implement strategies to teach them coping skills for depression, which is very appropriate for improving mental health [37, 39, 41, 45].

The impact that lifestyles had on students’ depression was evident since it was increased in the presence of toxic habits such as smoking. Likewise, internal feelings such as fear and stress, being only studying and having a close person infected also contributed to increasing depression. However, other behaviors such as e-health literacy were related to lower levels of depression [42, 48].

Some of the most striking results were that 80% of the students were concerned about the impact that e-learning would have on their ability to succeed academically. In fact, 62% of them were not confident that they would be able to cope with the workload during the pandemic or that they would have the appropriate technological means to carry out the pandemic. Among the main causes are lack of emotional support due to anxiety, financial difficulties, social isolation due to not spending time in classrooms with peers and teachers, and lack of preparation for the new way of teaching which was associated with a higher prevalence of depression among nursing students. However, it had no impact on self-esteem and anxiety [46, 47].

### 3.5 Adopted coping mechanisms

As we have seen, the prevalence of depression has been very high among students and almost half of them, 49.2%, did not present strategies to deal with mental health problems [43]. However, the strategies that reduced depression are described below.

Several authors show that high resilience was associated with less anxiety and stress, and therefore less depression. Similarly, a well-functioning and supportive family environment resulted in a lower risk of depression as lower levels could be observed among students who lived with their parents during confinement. Spiritual support was also seen as a coping mechanism for depression, as this feeling of connection to an external higher power seems to have helped reduce the prevalence of depression [38].

Mendez-Pinto et al., showed the important role that coexistence has in the levels of depression for nursing students during the COVI-19 pandemic since the data shown shows that 36.3% of those who suffered from depression lived with friends, while those who had another situation was 33.3%, those who lived with parents was 12.1% and those who lived alone was 7.6%. Therefore, living alone or with parents during the pandemic was protective for students [49].

In the study by Ozturk et al. [50], laughter therapy was implemented online with students to see how it affected their levels of stress, anxiety and depression. The sample was separated into two groups where one group received the therapy and the other did not. A reduction in depression was observed in students exposed to the laughter therapy compared to those not exposed. However, there was improvement in the other emotional problems.

Another coping mechanism students used to deal with the pandemic situation was to stay informed during the COVID-19 confinement. Those who chose to get information from social media reported significantly higher levels of stress, anxiety and depression, possibly related to information overload, sometimes even from dubious sources. However, students who chose to seek information from reliable sources such as the government or official media or the WHO were not as exposed to fake news, which clearly has a negative impact on mental health [37].

According to Kells et al. [44] 19.8% of nursing students reported moderate to severe depression. However, 54.4% of these students reported that the COVID-19 pandemic had reinforced their interest in pursuing nursing education. During the confinement, a sense of pride in the profession and in health professionals emerged among the students, which positively encouraged them to continue with their studies and not to drop out. This came about because they felt inspired and determined to help in the health crisis. The example of seeing strength, perseverance and teamwork reaffirmed the vocation to help others and increased the desire to be nurses in their own right. Therefore this motivation was an added coping method.

## 4. Discussion

**Fig 3:**
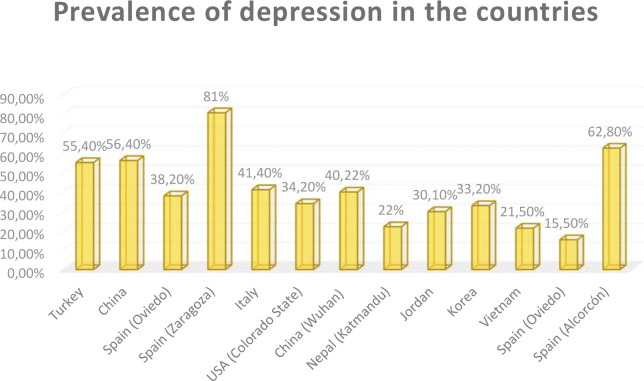
Prevalence of depression in nursing students during COVID19 distributed by country.

The variety of countries of origin is obvious from our results [37–50]. A clear relationship has been found between the country of residence of nursing students and the predisposition to suffer from depression. Prevalence values for this disorder range from 22.3% in countries such as Nepal [51] to 81% in Spain [52]. According to recent studies, the risk factors that predispose nursing students to suffer from depression are very heterogeneous. In Spain, an increase in depression was observed in comparison with other countries (Fig 3). This increase may have been caused by the way the pandemic developed, with a very high increase in patients with COVID-19, suspected cases and deaths during the different waves in the country. All of this obviously had an impact on a personal level, increasing concern, anxiety, stress and finally depression about the possibility of infecting themselves or their loved ones. This heterogeneity of the results is probably due to the way of dealing with the situation in each place [53].

The socio-demographic characteristics of the students are a very relevant topic. One characteristic analysed in the results is the influence of gender on depression [37, 38, 40, 44, 45, 50]. Regarding gender, there is controversy, since some affirm that men suffer more depression [54] and others believe that women [55]. In the study by Cam et al. [56], it was evident that being a woman contributed as a risk factor for suffering from depression and post-traumatic stress disorder during the COVID-19 pandemic. However, women represent 87.5% of nursing students and this may explain the higher prevalence in women. The difference between the sexes may be due to socio-cultural factors, as women in general tend to show their feelings more easily and suffer from problems of harassment or discrimination. This allows them to admit to problems related to anxiety and depression. Biological factors such as the involvement of hormones or genetic predisposition that makes women more vulnerable should also be considered [57–59]. With regard to age, there is little variation, although a relationship has also been established [40, 44]. Only some authors state that as age increases, the risk of suffering from depression is greater [60]. Younger students are considered a predictor for depression and may be associated with the disruption of classroom-based education, uncertainty about the academic path and career future of students, especially those who have just started their studies [61, 62]. University students have a high prevalence of this clinical picture although these figures cannot be used for diagnosis. This is due to the fact that various scales or screening instruments are used in the assessment of these symptoms. In addition, the tendency is to increase with age due to taking on new responsibilities or to decrease with age due to inexperience. Therefore, each student should be placed in his or her social context, as unemployment, violent conflicts, financial or marital problems may play a role.

Finally, it is important to highlight the influence of lifestyles [42, 48], living with others or not [38] and the coping strategies used during the pandemic [37, 39, 41, 45]. Personal care also influenced the suffering of depression. Lack of exercise [54] and being overweight [63] were related to this disorder among students. Regarding the family environment, the lack of support [54] and the poor economic situation contributed to the students suffering from depression [51]. A statistically significant correlation has been shown between cohabitation and depression, where depression decreases as the number of cohabitants increases [64]. However, it was shown that the prevalence in students who lived alone was lower (7.6%) compared to those who lived with their parents (12.1%). This can be explained by the fear of infecting family members, which was reduced by not residing with them and therefore the levels of depression were also lower [62].

From an academic point of view, concerns about work overload and the development of online classes were highlighted among nursing students [46, 47]. This is supported by studies showing higher levels of stress among nursing students compared to other students in other degree programmes [65]. The causes are often changes in the timetable [66], the teaching methodology [50] and obviously the fact of doing clinical placements during the COVID19 pandemic due to the fear that it produced [31, 32, 51, 52, 67]. Indeed, in the study by Browning et al. [68], students felt particularly demotivated, anxious and overwhelmed by the COVID-19 pandemic.

The lack of support and interpersonal relationships among students also affected their mental health. One of the main reasons was the increasing social distances between the population due to quarantine [37, 39]. This is because in the absence of interpersonal relationships, depression is more likely to increase [69]. In addition, students’ finances were found to be a predictor of psychological distress, since if they came from families with limited financial resources they expressed concern about the economic crisis, which was observed to have an impact on young people’s mental health [46, 47]. It is therefore natural to speak of a protective role of financial security against students’ psychological distress and various worries [70].

One of the coping mechanisms that best contributed to resilience was support from those living with the students [38, 49]. Several results showed the importance of including coping techniques for students during their academic day. Among the most effective coping mechanisms are resilience and spiritual support. Spiritual support arises from the human need to connect with a higher power, while resilience is the ability to adapt to stressful circumstances as was the case with the pandemic [71]. On the other hand, family support also had positive effects on reducing depression in nursing students as the lowest symptoms were associated with those living with their parents during confinement, although family functioning as a coping mechanism was not assessed. What is clear is that social distancing was a risk factor and being in close contact with family appears to be a protective factor, reducing levels of anxiety and depression [70].

With regard to laughter therapy as a mechanism to reduce depression, it was observed that students who underwent online laughter therapy during the pandemic were effective in reducing levels of depression [50]. This may be related to the introduction of more oxygen into the students’ bodies with deep breathing exercises, warm-ups and the lowering of cortisol. It is believed that during the COVID-19 pandemic, these online group activities such as laughter therapy created a relaxed environment in which to interact with peers, thereby improving depression [72].

On the other hand, students expressed concerns about infections, the shortage of personal protective equipment and the lethality of the virus [41, 45]. The pandemic affected the mental health of nurses, with nurses requiring supportive psychological therapy [73]. It is therefore not uncommon for student nurses to be affected as well. These circumstances aggravated and challenged the nursing students. Females had a higher perceived severity of infection and fear. Overall, it was related to lack of knowledge and this caused mental problems among the students. High knowledge about COVID-19 disease was shown to alleviate fear. It is therefore very important for nursing students to receive information and guidelines to decrease the fear of contagion as it improves mental health and coping. However, despite all this, they decided to continue their studies and increased their motivation due to the desire to help others, a strong motivation for most of them. The important work of nursing had an impact on the health of citizens and was visible in the media during the COVID-19 pandemic [74–76].

As mentioned above, social media has had a considerable impact on the information received by young people during the pandemic and has even influenced their mental health [37]. Images, videos and texts of various kinds can be easily accessed on social networks. Their dissemination is very simple, as it is enough to share them with contacts or even in profiles open to anyone who wants to follow them. During the pandemic, many movements and campaigns were created around the pandemic with various hashtags on both Instagram and Facebook. This caused, especially at the beginning of the confinement, a lot of stress and fear, which was reflected in the posts circulating on the internet [77]. It has been shown that when there is e-health literacy, it allows a healthy lifestyle and encourages prevention during the COVID-19 pandemic [78].

The study has some limitations that should be noted. With the exception of the clinical trial, most of the reviewed studies are observational/cross-sectional. Therefore, the level of evidence of these studies is generally low. In fact, this is the reason why more than one tool has been used during the process. However, these types of studies are suitable for analysing prevalence and relating variables and their integration into a systematic review, such as the one that has been carried out. Moreover, the countries of origin of the studies are very varied, as is the organisation of their education and health systems, so this should be taken into account when interpreting the data. It should also be noted that we have limited the chronological period from the beginning of the COVID-19 pandemic, so that other data on depression in students prior to that date have not been taken into account. Finally, the possible biases of the studies included in the review have to be taken into account, which we have tried to control by critically reading the studies prior to their inclusion. Above all, it is important to take into account possible publication bias, which we have tried to minimise by searching different databases with two independent reviewers.

Among the applications for clinical practice and future lines of research, it would be important to conduct primary studies on this disorder. These studies could opt for diverse methodological designs that deepen the understanding of depression in university students in general and nursing students in particular. This would make it possible to explore possible differences in prevalence according to the characteristics of the population groups and to plan coping strategies for the university population. This would ensure that future health professionals arrive to practice in the best possible conditions.

## 5. Conclusions

Amidst the COVID-19 pandemic, university nursing students, particularly women, have faced a heightened risk of experiencing depression and anxiety. This vulnerability has been exacerbated by widespread lockdowns and social isolation, with varying impacts based on geographic location and the severity of the outbreak in different countries.

Academic challenges have been significant, marked by an overwhelming workload and the transition to online teaching methods. As a result, students have employed a variety of coping mechanisms, both beneficial and less healthy, and have actively advocated for the necessary psychological support from their university institutions. However, there were unintended consequences such as a decrease in physical activity and sleep quality.

Economic disparities have further exacerbated negative mental health outcomes, with those with financial difficulties being more prone to depression. The role of social networks and information sources has been crucial in shaping the mental health of young individuals, although the prevalence of fake news has been a concerning factor. Emphasizing the importance of obtaining information from reliable sources, such as the Ministry of Health or the World Health Organization (WHO), is crucial in mitigating misinformation.

Despite the challenges, leaving nursing studies is not an option for these students. They draw motivation, vocation, and pride in their profession from media representations and the examples set by health professionals who have courageously fought against COVID-19, solidifying their commitment to making a positive impact and helping others. In the face of unprecedented challenges during the COVID-19 pandemic, nursing students exemplify resilience and unwavering dedication to their profession, drawing inspiration from the courageous efforts of healthcare professionals. Their commitment to making a positive impact and helping others remains steadfast despite the heightened risks and adversities they have encountered.

## Supporting information

S1 Checklist PRISMA 2020 checklist. (DOCX)

S1 Table Critical reading MMAT checklist [34]. (DOCX)
