# Supplementary material for: Depression in nursing students during the COVID-19 pandemic: Systematic review and meta-analysis
Source: PLoS One. 2024 Jul 24;19(7):e0304900. doi: 10.1371/journal.pone.0304900 (PMC11268638; doi:10.1371/journal.pone.0304900)
Supplement: S1 Table — (DOCX) [file pone.0304900.s002.docx]

Supplementary Table 1. Critical reading MMAT checklist (34)

| **AUTHOR AND YEAR (COUNTRY)** | | **Item 4.1** | **Item 4.2** | **Item 4.3** | **Item 4.4** | **Item 4.5** |
| --- | --- | --- | --- | --- | --- | --- |
| Alomari et al^46^, 2021 | | Y | Y | Y | Y | Y |
| Alsolais et al^45^, 2021 | | Y | Y | Y | CT | Y |
| Kells and Jennings^44^, 2023 | | Y | Y | Y | Y | Y |
| Kim et al^38^ , 2021 | | Y | Y | Y | Y | Y |
| Kwon^42^, 2023 | | Y | Y | Y | CT | Y |
| Losa-Iglesias et al^47^, 2023 | | Y | Y | Y | Y | Y |
| Mendez-Pinto et al^49^, 2023 | | Y | Y | Y | Y | Y |
| Mosteiro-Diaz et al^48,^ 2023 | Y | | Y | Y | Y | Y |
| Nguyen et al^43^, 2023 | | Y | Y | Y | CT | Y |
| Nihal Bostanci et al^37^, 2021 | | Y | Y | Y | Y | Y |
| Ozturk and Tekkas- Kerman^50^, 2022 | | Y | Y | Y | CT | Y |
| Patelarou et al^40^ , 2021 | | Y | Y | Y | Y | Y |
| Rayan^41^ , 2023 | | Y | Y | Y | CT | Y |
| Urban et al^39^, 2022 | | Y | Y | Y | Y | Y |

Note: N= No; Y =yes; CT = Can´t tell.
